# Validation of a transparent decision model to rate drug interactions

**DOI:** 10.1186/2050-6511-13-7

**Published:** 2012-08-20

**Authors:** Elmira Far, Ivanka Curkovic, Kelly Byrne, Malgorzata Roos, Isabelle Egloff, Michael Dietrich, Wilhelm Kirch, Gerd-A Kullak-Ublick, Marco Egbring

**Affiliations:** 1 Department of Clinical Pharmacology and Toxicology, University Hospital Zurich, Rämistrasse 100, 8091, Zurich, Switzerland; 2 Division of Biostatistics, ISPM, University Zurich, Hirschengraben 8, 8001, Zurich, Switzerland; 3 Department of Orthopaedic, Balgrist University Hospital, Forchstrasse 340, 8008, Zurich, Switzerland; 4 Institute of Clinical Pharmacology, Medical Faculty Technical University of Dresden, Fiedlerstrasse 27, D - 01307, Dresden, Germany

**Keywords:** Algorithm, Severity, Validation, Drug, Interaction, Decision, Model, Mmx, Epha.ch

## Abstract

**Background:**

Multiple databases provide ratings of drug-drug interactions. The ratings are often based on different criteria and lack background information on the decision making process. User acceptance of rating systems could be improved by providing a transparent decision path for each category.

**Methods:**

We rated 200 randomly selected potential drug-drug interactions by a transparent decision model developed by our team. The cases were generated from ward round observations and physicians’ queries from an outpatient setting. We compared our ratings to those assigned by a senior clinical pharmacologist and by a standard interaction database, and thus validated the model.

**Results:**

The decision model rated consistently with the standard database and the pharmacologist in 94 and 156 cases, respectively. In two cases the model decision required correction. Following removal of systematic model construction differences, the DM was fully consistent with other rating systems.

**Conclusion:**

The decision model reproducibly rates interactions and elucidates systematic differences. We propose to supply validated decision paths alongside the interaction rating to improve comprehensibility and to enable physicians to interpret the ratings in a clinical context.

## Background

The management of adverse drug events (ADEs) is an important issue in healthcare [1]. While some ADEs are unpredictable (e.g. anaphylaxis), ADEs caused by drug-drug interactions (DDI) are likely to be preventable [2]. Nevertheless, DDIs continue to present a major problem in medical treatment. One Swiss study estimated that 17% of all ADEs occurring in hospitalized patients are provoked by DDIs [3], while a Dutch study found that 28% of patients admitted to the hospital experienced at least one DDI [4]. Clinical decision support software (CDSS) has been used as a supportive measure to improve medication safety [5,6]. The information provided by CDSS focuses on management advice rather than alerts, since more prevalent alerts may dominate less common but equally dangerous ones [4].

In the past, DDIs were classified according to their potential severity e.g. minor, moderate, or major. In 2001 a new management-oriented approach to DDI classification was advanced by Hansten and Horn [7]. More than 75% of majorly severe interactions are considered manageable [8]; therefore this approach seems reasonable. Recently, a separate group in our department developed ZHIAS (Zurich Interaction System), an extension of the clinical management approach, which is based on Operational Classification of Drug Interactions (ORCA) [9,10]. Another management-oriented classification system is based on types of adverse drug reactions [8]. Even with multiple classifications being available, the assessment of DDIs depends on both the experience and the interpretation of the assessor as well as the sources of information used in the assessment [11]. The discrepancies between different DDI ratings are well-documented [7,12-14]. No two DDI databases use the same set of criteria to assign severity ratings [15]. For example, the assigned interaction severity between alprazolam and digoxin ranges from “no interaction” to “major interaction”, depending on database [16-19]. It remains unclear whether these rating discrepancies arise from inconsistent study results or from the use of different DDI classification algorithms. One case report and one study showed that plasma digoxin concentrations significantly increase in the presence of alprazolam [20]. A separate study involving healthy volunteers reported no clinically relevant change in digoxin plasma concentrations [21]. In the past 30 years, more than 15,000 papers on DDIs have been published [7]. The problem we face today is not the lack of information on DDIs or the type of classification, but the incompatibility of DDI rating systems. Alerts are often disregarded by physicians, if background information on the decision layer and practical management recommendations are lacking [22,23]. In order to increase user acceptance, the DDI rating must be consistent and comprehensible, and the decision model must be transparent [24].

To improve rating comprehensibility, we developed a transparent decision model (DM) to rate drug interactions. The model is based on previous research by van Roon and colleagues [25]. The aim of our current research is to validate the transparent decision model in terms of reproducibility and identification of systematic differences between DDI ratings.

## Methods

### Design of decision model

**Figure 1:**
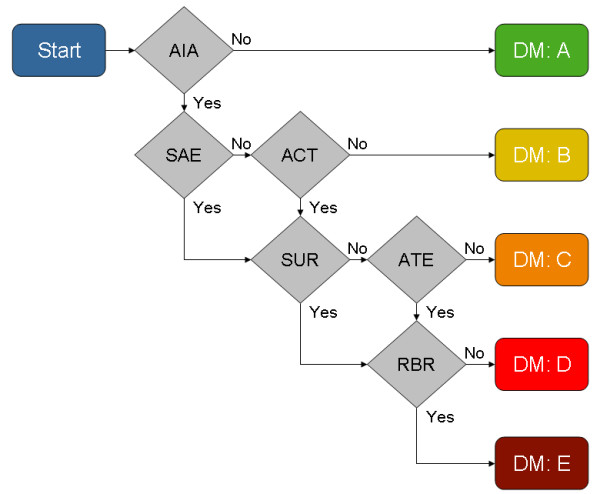
**Visualisation of proposed action-oriented decision model to rate drug-drug interactions.** The six question sets relate to: AIA (apparent interaction), SAE (serious adverse event), ACT (action), SUR (surveillance), ATE (alternative), RBR (risk-benefit ratio). Five possible ratings are DM: **A** (no action required), DM: **B** (precautionary measures), DM: **C** (clinical monitoring), DM: **D** (avoid) and DM: **E** (contraindicated).

In designing the DM, we developed a list of binary questions which we considered would impact on the interaction rating. Similar questions were constructed iteratively, and six sets of clinically relevant questions were ultimately retained. The questions were evaluated regarding their relevance to a robust and comprehensible DDI rating system. The sequential order of the six binary questions (see Figure 1) was permuted by a review team consisting of one pharmacist, two clinical pharmacologists and one physician, until consensus regarding the rating outcome of the DM was achieved.

The six question sets are outlined as follows:

1. Apparent interaction (AIA) comprised two sub-questions:

Only one “yes” answer is required to progress down the decision path to the next question.

a) *Has this interaction been described in the scientific literature (e.g. credible clinical studies and credible case reports)?*

b) *Can one postulate a plausible, hypothetical mechanism of pathogenic interaction?*

2. Serious adverse event (SAE) inquires into the clinical severity of the interaction: *Is there an increased risk for the occurrence of an SAE within the normal patient population?*

3. Action (ACT) determines whether medical intervention is necessary: *Does the interaction outcome necessitate medical intervention, other than simple precautionary measures?*

4. Surveillance (SUR) ascertains whether the consequences of the interaction can be easily monitored: *Is the interaction risk difficult to assess in an out-patient setting and within a short time-frame?*

5. Alternative (ATE) questions whether a safer alternative to either one of the drugs exists. It comprises two sub-questions.

Both questions must be answered “yes” in order to proceed to the final step of the decision model.

a) *Does a suitable alternative exist (within the same ATC category), which carries a lower potential for interaction?*

b) *Are credible dose adjustment guidelines unavailable?*

6. Risk-benefit ratio (RBR): *Does the risk outweigh the potential benefit?*

The DM presents 13 possible decision paths leading to 5 possible interaction ratings: DM: A (no action required), DM: B (precautionary measures), DM: C (clinical monitoring), DM: D (avoid) and DM: E (contraindicated). For statistical analysis numbers 1 up to 5 were assigned to the ratings. The ratings are defined to avoid ambiguity and are based on clinical management. A rating of DM: A indicates that co-administration is safe, based on currently available scientific data. When an interaction is rated DM: B, precautionary monitoring for unusual side effects is sufficient. DM: C signifies that, although no alternative therapies are available, the likely effect of the interaction is easily monitored. Necessary medical action will be guided by the relevant published medical guidelines. DM: D indicates that co-administration should be avoided and only undertaken when deemed imperative. DM: E states clearly that the drugs must not be co-administered in any clinical situation. The interaction ratings were standardized to ensure consistency in rating outcomes by different physicians/pharmacists. The DDI rating was designed for integration into a network of additional decision support systems, such as patient-specific risk factors (e.g. old age, obesity, or renal insufficiency) or drug-disease state contraindications, whereas the DM refers to the low-risk normal population. A serious adverse event is defined as a life-threatening or debilitating event, resulting in death, inpatient hospitalization or prolongation of existing hospitalization, or persistent or significant disability/incapacity. Risk/benefit defines the balance between the effectiveness of a medicine and the risk of harm as specified by the World Health Organization Uppsala Monitoring Centre (WHO-UMC) in Sweden.

### Other ratings

One of our assessors, a clinical pharmacologist, classified DDIs into five categories, namely: “no interaction”, "minor", "moderate", "major" and "contraindicated", based on her personal clinical experience and interpretation of the available literature relating to drug interactions. The Micromedex DrugDex (MMX) database classifies DDIs as "unknown", "minor", "moderate", "major" or "contraindicated". MMX also estimates the quality of DDI documentation, rating it as either "excellent", "good", "fair" or "unknown".

### Validation of decision model

In our study we randomly selected 200 potential drug interactions and compared the individual rating outcomes generated by three different rating methods. Clinical relevance of the drug interactions was assessed from queries received at the Department of Clinical Pharmacology and Toxicology at the University Hospital in Zurich, raised by pharmacists and physicians in primary and secondary care and from ward rounds at the University Hospital. In the first rating method, one pharmacist applied our DM to manually rate the 200 interactions. The ratings were then reviewed and revised for plausibility by a team comprising two clinical pharmacologists and one physician. The second rating was performed by an independent senior clinical pharmacologist who was blinded with respect to the DM and who assigned each interaction rating based on her clinical experience and knowledge. The clinical pharmacologist was not permitted use of an interaction database, but was allowed access to available scientific sources such as PubMed database, Excerpta Medica database (Embase), European Public Assessment Reports (EPARs) and summary of product characteristics. The same information sources were accessible to the pharmacist. In the third rating method, a physician rated the 200 interactions using the commercially available MMX database [16].

### Statistical methods

The concordance between all three ratings was determined using cross-tables, together with ordinary and weighted Cohen’s Kappa coefficients. Cohen’s Kappa measures the extent to which any two rating systems agree by chance alone. It ranges from zero (agreement no better than chance) to one (perfect agreement). In the tables, values adjacent to the diagonal (ratings differing by a single category) are considered less serious than deviations of two or more categories. Cohen’s Kappa evaluates inter-rater agreement as follows: 0.01–0.2 slight agreement; 0.21–0.40 fair agreement; 0.41–0.60 moderate agreement; 0.61–0.80 substantial agreement and 0.81–1 perfect agreement [26]. To identify systematic differences between the rating systems, Bland–Altman plots, which illustrate agreement limits, were constructed. Identified systematic differences were reviewed individually by the aforementioned review team and were excluded from further analysis. The relative frequencies and confidence intervals of the remaining disagreements were determined by the Wilson method [27].

## Results

The pharmacist, physician and the clinical pharmacologist independently assessed all cases of potential drug interactions (n = 200). 62 of the interactions yielded no information from MMX regarding possible DDIs. The ratings evaluated by the pharmacist and the clinical pharmacologist ranged from DM: B (precautionary measures) to DM: E (contraindicated).

### Concordance

Agreement between the DM and the clinical pharmacologist was high, with a ordinary Kappa coefficient of 0.692 (95% CI [0.611, 0.744]) and weighted Kappa of 0.805 (95% CI [0.747, 0.863]). Agreement between the DM and MMX was fair with a ordinary Kappa coefficient of 0.315 (95% CI [0.233, 0.397]) and weighted Kappa of 0.363 (95% CI [0.276, 0.449]). The DM was concordant with the clinical pharmacologist and with MMX in 156 (78% (95% CI [72, 83])) and 94 cases (47% (95% CI [40, 54])), respectively. Likewise the clinical pharmacologist and MMX agreed in 89 (45% (95% CI [38, 51])) of the 200 interaction cases. Tables 1 and 2 show the DDI cross-ratings between DM and clinical pharmacologist and DM and MMX, respectively.

**Table 1:** Cross correlation of drug-drug interaction ratings for clinically identified cases (n = 200) between the proposed decision model (DM) and a clinical pharmacologist

		**Clinical Pharmacologist**					
		**A**	**B**	**C**	**D**	**E**	**Total**
DM	A	18	3	0	0	0	21
	B	2	10	0	0	0	12
	C	0	0	49	5	0	54
	D	0	1	**30**	60	2	93
	E	0	0	0	1	19	20
	**Total**	20	14	79	66	21	**200**

Ratings are A (no action required), B (precautionary measures), C (clinical monitoring), D (avoid) and E (contraindicated). Systematic differences between ratings are highlighted.

**Table 2:** Cross correlation of drug-drug interaction ratings for clinically identified cases (n = 200) between the proposed decision model (DM) and Micromedex (MMX)

		**MMX**					
		**A**	**B**	**C**	**D**	**E**	**Total**
**DM**	A	21	0	0	0	0	21
	B	**8**	0	4	0	0	12
	C	**18**	4	26	6	0	54
	D	**32**	1	**25**	34	1	93
	E	**4**	0	0	3	13	20
	**Total**	83	5	55	43	14	**200**

Ratings are A (no action required), B (precautionary measures), C (clinical monitoring), D (avoid) and E (contraindicated). The rating X from MMX has been labeled E. Missing ratings from MMX have been labeled A. Systematic differences between ratings are highlighted.

### Divergence

We corrected the rating of the pharmacist in two cases, where the DM was applied incorrectly. The application error rate occurred in 1% of all 20 cases (95% CI [0, 3]). The first error, in the assessment of roxithromycin and simvastatin co-administration, was caused by incorrect interpretation of the DM question. The pharmacist applied the serious adverse events (SAEs) question to the “at-risk” population instead of to the “normal patient” population. Therefore the rating of DM: D (avoid) assigned to this interaction by the pharmacist, required correction to DM: B (precautionary measures). No further information about this rating was extracted from MMX, so a third rating was unavailable for comparison. The second error regarded the combination of atenolol and bupropion. The pharmacist did not use all available information to rate the interaction and in particular did not consider that co-administration can induce blood pressure changes, and thus may alter the effect of atenolol. Therefore the rating of DM: A (no action required) assigned to this interaction by the pharmacist, required correction to DM: B (precautionary measures).

### Systematic difference

**Figure 2:**
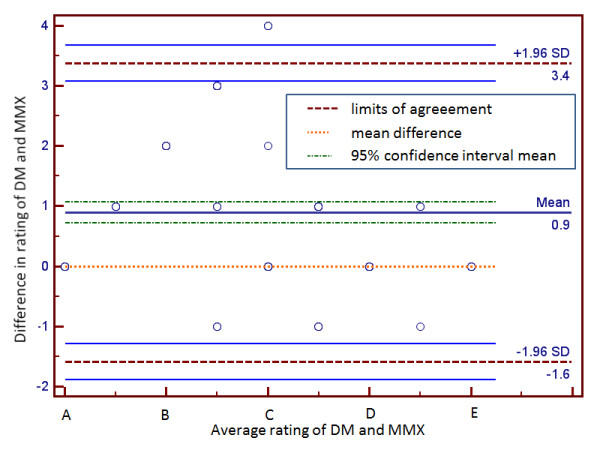
**Bland-Altman plot of differences in ratings assigned to clinically identified drug-drug interactions (n = 200) by the proposed decision model (DM) and Micromedex (MMX).** Data includes those interactions for which MMX had no rating (n = 62) as highlighted in cells (A,B),(A,C),(A,D) and (A,E) from Table 2.

Systematic differences between the ratings of DM and MMX are displayed as a Bland–Altman plot in Figure 2. The mean difference is 0.9 and perfect agreement (zero) lies outside the confidence interval. The rankings differed by up to three classification categories. The limits of agreement were [−1.6, 3.4], indicating that the DM tends to rate a higher severity. Not shown are the systematic differences for clinical pharmacologist versus MMX (mean difference: 0.8, limits of agreement [−1.5, 3.1]), and DM versus clinical pharmacologist (mean difference: 0.13, limits of agreement [−0.8, 1.05]).

**Figure 3:**
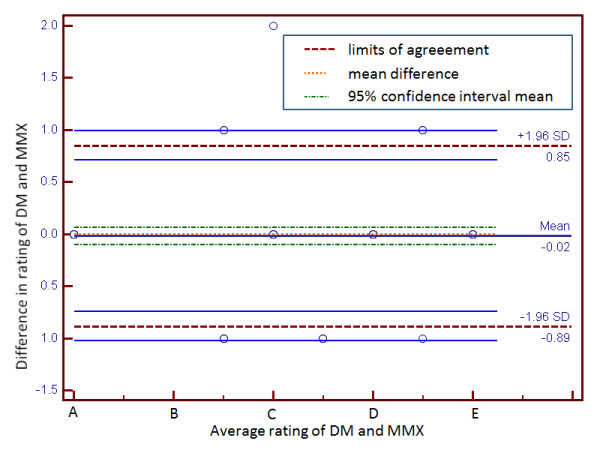
**Bland-Altman plot of differences in ratings assigned to clinically identified drug-drug interactions (n = 113) by the proposed decision model (DM) and Micromedex (MMX).** Data is based on 200 drug-drug interactions, but excludes those interactions for which MMX had no rating (n = 62) and those ratings with systematic differences (n = 25) (highlighted cells in Table 2).

Systematic-difference based disagreements in DM versus MMX and DM versus clinical pharmacologist assessments were excluded from further analysis. The corresponding cells are highlighted in Tables 1 and 2. Figure 3 shows the Bland–Altman plot of the remaining data set for DM and MMX. The mean difference decreased to −0.02, statistically the same as perfect agreement, while limits of agreement narrowed down to [−0.89, 0.85]. The rankings differed by at most one rating.

The remaining 14 (of the 200 ratings) disagreed between the DM and clinical pharmacologist for reasons not explained by systematic differences (these non-systematic discrepancies account for 7% (95% CI [4,11]) of all ratings). The remaining 19 non-systematic disagreements between DM and MMX constitute 9.5% (95%CI: [6,14]).

## Discussion

We evaluated a transparent decision model that reproducibly rates drug interactions and identifies systematic rating discrepancies. Altman [26] suggests that kappa is the appropriate means of judging agreement or reproducibility between classification categories obtained by two different rating methods and is supported by the higher weighted Kappa values, which strengthened the approach in the present study. No systematic differences showed up on the Bland–Altman plot of DM versus MMX, following removal of the systematic differences. Divergence in decision making remains an issue and review of certain cases is unavoidable. The review time, however, decreases as a result of the standardization. When comparing two ratings, our visualization of the decision path enables rapid comprehension of one side of the differences [28], thus clarifying (at least partially) the rating discrepancies. Such transparency improves the clinical value of the interpretation of the rating [29,30]. To our knowledge, we publish the first visualized decision model that is comparable with other ratings. Previously published ratings, though based on expert group decisions, are not guided by specified rules of an algorithm. The output of the decision model, corrected for systematic differences between rating systems, closely resembles that of other ratings. To illustrate the systematic nature of these differences, we summarize the most important ones (highlighted in the cross tables) below.

### Systematic differences

If more than simple precautionary measures are required in first line therapy, or if complex monitoring of a likely side-effect is required, we assume that a suitable drug alternative precludes co-administration, because the latter disproportionately raises patient risk or health care costs. This explains why DM rated 30 cases of higher severity than the clinical pharmacologist (Table 1) and 25 cases of higher severity than MMX (Table 2).

Interactions requiring complex monitoring were rated of higher severity by DM than either the clinical pharmacologist (DM rated 18 of 30 cases more severely) or MMX (DM rated 21 of 25 cases more severely). (i) The clinical pharmacologist assigned a rating of C (“moderate”) to the combination of citalopram and tramadol, whereas both DM and MMX recommended avoiding this combination (ratings: DM: D and “major”, respectively), since co-administration increases the risk of serotonin toxicity. Monitoring for SAEs such as hyperreflexia, CNS symptoms, myoclonus, sweating and hyperthermia is imperative and is complex in an outpatient setting. (ii) Risk of amiodarone and phenytoin co-administration was rated C (“moderate”) by MMX and C (“precautionary measures”) by the clinical pharmacologist. The DM assigned a rating of D (“avoid”), since amiodarone concentrations in plasma may be reduced to as low as 30% in the presence of phenytoin. This effect can occur several weeks into phenytoin therapy, therefore amiodarone concentrations must be monitored for several weeks to enable dose adjustment. Furthermore, phenytoin toxicity can occur and surveillance requires considerable effort. (iii) Co-administration of duloxetine and amitriptyline increases the risk of anticholinergic or serotonin syndrome and may lead to elevated amitriptyline plasma concentrations. Because of the complex clinical surveillance required, this interaction was rated D by the DM, whereas MMX assigned a C rating.

The inclusion of suitable treatment alternatives in the decision process caused DM to rate an interaction more severely than the clinical pharmacologist in 12 of 30 cases, and more severely than MMX in 4 of 25 cases. (i) Co-administration of digoxin and alprazolam was rated C by the clinical pharmacologist, since alprazolam interferes with digoxin levels and therefore requires drug concentration monitoring at the initiation and discontinuation of alprazolam therapy. The DM rated this interaction as D, because a suitable alternative (lorazepam) exists. (ii) MMX rated the combination of midazolam and phenytoin as “moderate”. Although the co-presence of phenytoin depresses midazolam levels, alternative benzodiazepines are available which carry a lower potential for interaction.

In one case, a rating discrepancy of two categories was found (the drug combination was rated B by MMX and D by DM). The drugs in question were fluconazole and fluvastatin, for which co-administration increases the risk of severe myopathy while an alternative to fluvastatin exists.

### Study limitations

This study focused solely on the decision making process, and the positive contribution of the rating output to medical therapy was not evaluated. Although every attempt has been made to ensure that the categories are objective (i.e. they represent a consensus between four clinical specialists in three different fields), they are nonetheless subject to user interpretation and should not be regarded as a “gold standard”, but as an approach to standardize ratings with defined rules. We hope that publication of this decision model will stimulate other groups to test the models’ reproducibility. The feasibility of the decision model to illustrate system differences has been tested with a single database, MMX. In future, the DM may elucidate systematic differences between other rating discrepancies reported in the literature [11,13,14]. Concordance between the DM and expert assessment has been validated by only one pharmacist from our group.

The agreement between DM and MMX was evaluated as “fair”, which can be explained partly by systematic differences in 25 cases, but which must also consider the missing information from MMX in 62 cases. The omission of information in MMX regarding a specific drug combination cannot be considered as the absence of a DDI. Therefore our database distinguishes between missing information and a safe combination (DM: A). No information was yielded by MMX for the following complications of drug co-administration. (i) The combination of phenobarbital and acetaminophen increases the risk of hepatotoxicity. (ii) The concurrent use of phenobarbital and mirtazepine may inhibit mirtazepine efficacy and therefore requires clinical monitoring. (iii) Duloxetine increases the area under the plasma concentration time curve (AUC) of metoprolol 1.8-fold. As a result, blood pressure and heart rate monitoring are required, particularly at the start and cessation of duloxetine therapy. Drugs that are used in Europe but not in the U.S. explain a portion of the missing data.

## Conclusions

The decision model reproducibly rates interactions and identifies systematic differences. Ratings are based on critical indicators of clinical significance, namely; the risk of an SAE, the extent of medical intervention required, the clinical surveillance required, the existence of a safer alternative and the risk-benefit ratio. The decision model is consistent with other rating systems, following removal of systematic differences between methods. We propose to supply the decision path alongside the interaction rating, to facilitate rating comprehensibility and to assess mortality and morbidity rates in a clinical setting. If factors such as length of hospital stay or risk of complications are improved by using the model, then the model represents a significant advance over existing models.

## Abbreviations

ADE: Adverse drug event; DDI: Drug-drug interaction; DM: Decision model; MMX: Micromedex DrugDex.

## Competing interests

Gerd Kullak-Ublick, Michael Dietrich and Marco Egbring are shareholders of the spin-off EPha.ch, which develops prescribing services. The cases in this publication have been included in the interaction database, which is published under a Creative Commons Attribution-Share Alike 3.0 Unported License. The other authors declare that they have no competing interests.

## Authors’ contributions

EF and IC performed the study, analyzed the data, discussed the results and drafted the manuscript. EF and IC contributed equally. KB helped to analyze the data and revised the manuscript. MR analyzed the statistical data and revised the appropriate paragraphs. IE participated in the design of the study and collected data. MD drafted the concept and revised the manuscript. WK provided valuable external expertise regarding the development. GK participated in the design and coordination of the study and revised the manuscript. ME designed the concept, analyzed data statistically and participated in the draft and revision of the manuscript. All authors read and gave final approval of the submitted version of the manuscript.

## Pre-publication history

The pre-publication history for this paper can be accessed here:

http://www.biomedcentral.com/2050-6511/13/7/prepub
